# Are prehospital airway management resources compatible with difficult airway algorithms? A nationwide cross-sectional study of helicopter emergency medical services in Japan

**DOI:** 10.1007/s00540-015-2124-7

**Published:** 2015-12-29

**Authors:** Yuko Ono, Kazuaki Shinohara, Aya Goto, Tetsuhiro Yano, Lubna Sato, Hiroyuki Miyazaki, Jiro Shimada, Choichiro Tase

**Affiliations:** Emergency and Critical Care Medical Center, Fukushima Medical University Hospital, 1 Hikarigaoka, Fukushima, 960-1295 Japan; Department of Anesthesiology, Ohta General Hospital Foundation, Ohta Nishinouchi Hospital, Koriyama, Japan; Department of Public Health, School of Medicine, Fukushima Medical University, Fukushima, Japan

**Keywords:** Airway equipment, Supraglottic airway device, Difficult airway, Prehospital endotracheal intubation, Surgical airway equipment

## Abstract

**Purpose:**

Immediate access to the equipment required for difficult airway management (DAM) is vital. However, in Japan, data are scarce regarding the availability of DAM resources in prehospital settings. The purpose of this study was to determine whether Japanese helicopter emergency medical services (HEMS) are adequately equipped to comply with the DAM algorithms of Japanese and American professional anesthesiology societies.

**Methods:**

This nationwide cross-sectional study was conducted in May 2015. Base hospitals of HEMS were mailed a questionnaire about their airway management equipment and back-up personnel. Outcome measures were (1) call for help, (2) supraglottic airway device (SGA) insertion, (3) verification of tube placement using capnometry, and (4) the establishment of surgical airways, all of which have been endorsed in various airway management guidelines. The criteria defining feasibility were the availability of (1) more than one physician, (2) SGA, (3) capnometry, and (4) a surgical airway device in the prehospital setting.

**Results:**

Of the 45 HEMS base hospitals questioned, 42 (93.3 %) returned completed questionnaires. A surgical airway was practicable by all HEMS. However, in the prehospital setting, back-up assistance was available in 14.3 %, SGA in 16.7 %, and capnometry in 66.7 %. No HEMS was capable of all four steps.

**Conclusion:**

In Japan, compliance with standard airway management algorithms in prehospital settings remains difficult because of the limited availability of alternative ventilation equipment and back-up personnel. Prehospital health care providers need to consider the risks and benefits of performing endotracheal intubation in environments not conducive to the success of this procedure.

**Electronic supplementary material:**

The online version of this article (doi:10.1007/s00540-015-2124-7) contains supplementary material, which is available to authorized users.

## Introduction

**Fig. 1:**
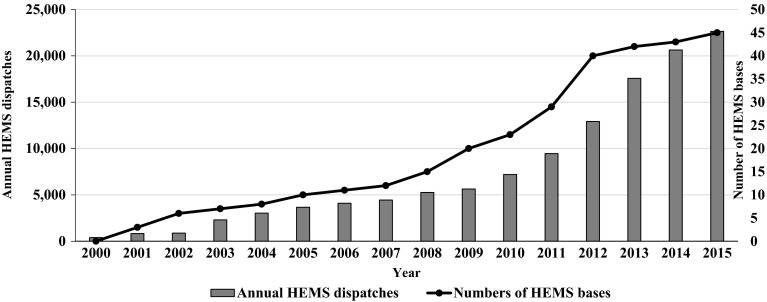
Growth of helicopter emergency medical services (HEMS) in Japan. The data were provided by the Japanese Society for Aeromedical Services, and the Emergency Medical Network of Helicopter and Hospital (HEM-Net)

Helicopter emergency medical services (HEMS) have been implemented in Japan since 2001 [1], with recent rapid increases in their use (Fig. 1). Annual HEMS dispatches exceeded 20,000 in 2013 (Fig. 1), and the number continues to rise [data kindly provided by the Japanese Society for Aeromedical Services, and the Emergency Medical Network of Helicopter and Hospital (HEM-Net)]. After the major earthquake in eastern Japan in 2011, HEMS played a crucial role in disaster-stricken areas by providing triage, treatment, emergency care, and transportation [2]. With the rapid growth of HEMS in Japan and the improved response to catastrophes such as earthquakes, prehospital endotracheal intubation (ETI) has become much more common.

ETI outside the hospital is challenging even for experienced providers. The rate of difficult ETI in prehospital settings ranges from 6.0–17.7 % [3–6], which is much higher than the rate in the hospital operating room [4]. Severe ETI-related complications, including severe hypoxia, esophageal intubation, aspiration, and cardiac arrest, are likely to occur in association with difficult airway management (DAM) [7–9]. Although the limited resources of prehospital settings are in part responsible for these difficulties [10], data are scarce regarding the availability of airway equipment, alternative ventilation devices, and drugs and the capabilities of care providers in Japanese prehospital environments.

Airway management algorithms have been advocated by the Japanese Society of Anesthesiologists (JSA) [11], the American Society of Anesthesiologists (ASA) [12], and by the Difficult Airway Society (DAS) [13]. These guidelines underlie the standards and principles that apply to the treatment of a patient with a difficult airway, not only regarding the induction of anesthesia but also for any other situation arising in the emergency department and prehospital setting. Their four key steps are [11–13] (1) call for help if any problems have occurred; if mask ventilation is not adequate, consider (2) supraglottic airway device (SGA) insertion; (3) confirmation of ETI using capnometry; and (4) establish a surgical airway if a ‘cannot ventilate, cannot intubate’ (CVCI) situation is encountered. The immediate availability of back-up staff and of proper DAM equipment, including SGA, capnometry, and a surgical airway device, is therefore indispensable. The authors of a report based on a national survey carried out in the UK concluded that, regardless of the location, DAM equipment should be consistent with that in the hospital operating room [14]. In fact, several studies have proposed that ETI in the prehospital setting should be performed according to the same standards that apply in the hospital [15–17]. However, whether prehospital airway management resources in Japan are compatible with the standards established in the DAM guidelines [11–13] has not been comprehensively evaluated.

Therefore, by conducting a national survey of HEMS, we sought to determine (1) the availability of airway devices, alternative ventilation, ETI confirmation equipment, drugs, and specialist care providers and (2) whether these resources comply with the JSA, ASA, and DAS airway management algorithms [11–13].

## Materials and methods

### Study design and sites

This cross-sectional study was conducted from May to July 2015. After approval by the institutional review boards of Fukushima Medical University (no. 2276), self-administered questionnaires were mailed to all HEMS base hospitals (45 bases in 37 prefectures) registered in HEM-Net. A complete list of these hospitals is available at the HEM-Net home page: http://www.hemnet.jp/english/where/index.html (accessed 22 October 2015).

### Survey items

When selecting items in the questionnaire, we referred to previous studies conducted in other countries and addressing similar (prehospital settings [18–20], emergency departments [21–23]) as well as different (obstetric units [24–26]) settings. We then circulated drafts among survey team members consisting of an epidemiologist, anesthesiologists, and physicians and nurses specializing in emergency medicine to finalize.

These survey items consisted of (1) basic information regarding the numbers of HEMS dispatches in 2014 and hospital beds, and the prehospital availability of the following materials—(2) direct laryngoscope and adjunct equipment (curved blade, straight blade, McCoy laryngoscope, stylet and gum elastic bougie); (3) alternative intubation equipment (rigid video laryngoscope, flexible fiber scope, retrograde intubation kit, and surgical airway equipment); (4) alternative ventilation equipment (SGA, oral and nasal airways); (5) device to confirm ETI (capnometry, esophageal detector); (6) a packaged unit containing the items listed in (2)–(4); and (7) drugs to facilitate ETI and reversal agents (analgesics, sedative, neuromuscular blocking agents [NMBAs], sugammadex, flumazenil and naloxone). In addition, information about (8) the prehospital care providers (number of prehospital physicians, nurses and on-the-job trainees) usually on board the emergency helicopters and (9) the board certifications of the prehospital physicians and nurses was obtained. Among these items, in (6)–(8) and in (9), the board certification of nurses had not been included in previous studies [18–26]. However, it was included in this study after discussion and mutual agreement among the authors. An English version of the Japanese questionnaire used in this study is available in the supplementary material. The questionnaire also queried the availability of direct laryngoscopes and alternative ventilation equipment in pediatric sizes. The product name of the rigid video laryngoscopes used was also requested. The contents of the packaging unit were determined according to the airway management guidelines of the JSA [11] and ASA [12]. Packaging was graded as complete, partial, and none. If SGA was available, its inclusion of an intubating laryngeal mask was determined. Surgical airway equipment was categorized as a cricothyroidotomy kit or a set containing a scalpel and hemostat. Board-certified physicians and nurses were defined based on the criteria of the Japanese Medical Specialty Board (http://www.japan-senmon-i.jp/, in Japanese, accessed 22 October 2015) and Japanese Nursing Association (http://nintei.nurse.or.jp/nursing/qualification/cn, in Japanese, accessed 22 October 2015), respectively. HEMS base hospitals that did not respond to the initial survey were sent a repeat mailing.

### Outcome measures

The JSA airway management algorithms [11], ASA DAM guideline [12], and DAS guideline [13] commonly endorse the following four steps in the treatment of an airway—(1) call for help if difficulties are encountered; (2) attempt SGA insertion if mask ventilation is not adequate; (3) use of capnometry to confirm correct endotracheal tube placement; and (4) establish a surgical airway if a CVCI situation has occurred. Outcome measures in this study included the feasibility of these four steps in the prehospital settings. ‘Call for help' was deemed feasible if more than one physician was usually on board. This is because, in Japan, only physicians are permitted to perform ETI and SGA insertion. On-the-job medical trainees were not regarded as physicians because they could have been staff-level physicians, junior residents, nurses, or paramedics. ‘SGA insertion' and ‘confirmation of ETI using capnometry' were presumed possible if the respective devices were carried on board. ‘Surgical airway' was deemed practicable if a cricothyroidotomy kit or a scalpel and hemostat were available on board. All outcome measures were defined by mutual consent among the five authors (YO, KS, AG, JS, and CT), which included three board-certified anesthesiologists.

### Statistical analysis

First, all survey items were evaluated using descriptive statistics. Second, the association between the feasibility of the four steps and annual HEMS dispatches, regions, and foundation date were analyzed using Fisher’s exact test. For HEMS dispatches, the values were dichotomized using the median. Regions were divided into east (Hokkaido, Tohoku, Kanto/Koshin, Hokuriku, Tokai) and west (Kinki, Chugoku, Shikoku, North and South Kyushu, Okinawa) according to the classification of the Japanese meteorological agency (http://www.jma.go.jp/jma/indexe.html, accessed 22 October 2015). The foundation date was divided into an early phase (2001–2008) and a late phase (2008–2015). All statistical analyses were performed using IBM SPSS Statistics for Windows, version 21.0 (IBM Corp., Armonk, NY, USA). A *p* value < 0.05 was considered to indicate statistical significance.

## Results

**Fig. 2:**
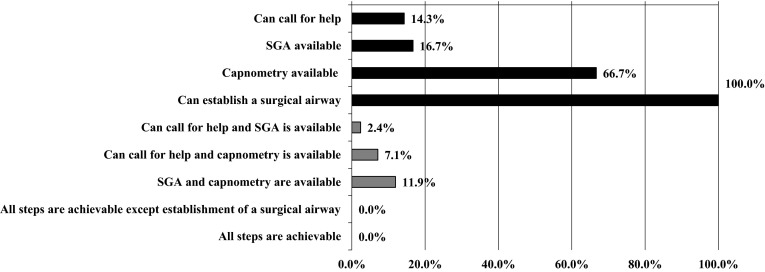
Availability in Japanese helicopter emergency medical services of the difficult airway management resources specified in the JSA, ASA, and DAS airway management algorithms. *ASA* American Society of Anesthesiologists, *DAS* Difficult Airway Society, *JSA* Japanese Society of Anesthesiologists, *SGA* supraglottic airway device

Of the 45 HEMS base hospitals, 42 returned the completed questionnaire (response rate = 93.3 %). None of these hospitals were excluded because of incomplete responses. The median number of annual HEMS dispatches was 447 (interquartile range 366–550); the median number of hospital beds was 653 (interquartile range 579–768). Table 1 summarizes the airway equipment available in Japanese HEMS. Among the HEMS bases that responded, only seven (16.7 %) had a SGA, five (11.9 %) of which also carried a pediatric-sized device. Capnometry was available in 28 (66.7 %) HEMS. All bases possessed a surgical airway device, either a cricothyroidotomy kit (61.9 %) or scalpel and hemostat (38.1 %). Table 2 lists the drugs available to facilitate ETI in prehospital settings. None of the HEMS had depolarizing NMBAs; 34 (81.0 %) had at least one non-depolarizing NMBA, 5 (11.9 %) had sugammadex, and 8 (19.0 %) did not have any type of NMBA. Table 3 provides information on the prehospital care providers. Two physicians were usually on board at six (14.3 %) bases. Of 347 attending physicians at all bases, the most common board certification was emergency medicine (75.8 %), followed by general surgery (15.6 %). Board-certified anesthesiologists comprised 10.1 % of all prehospital physicians. Figure 2 shows the availability in Japanese HEMS of the DAM resources specified in the JSA, ASA, and DAS algorithms. According to our feasibility definitions, ‘surgical airway’ was deemed attainable in all bases, ‘call for help’ in 14.3 %, ‘SGA insertion’ in 16.7 % (11.9 % in pediatric cases), and ‘confirmation of ETI using capnometry' in 66.7 %. There were no bases in which all steps were deemed achievable in the prehospital setting. Table 4 shows the associations between the feasibility of airway management guidelines and annual dispatches, region, and the foundation dates of the HEMS surveyed. None of the associations were of statistical significance.

**Table 1:** Airway equipment at 42 Japanese helicopter emergency medical services (HEMS)

Equipment item	*N* (%)
Direct laryngoscope and adjunct^a^	
Curved laryngoscope blade (Macintosh type)	42 (100)
Pediatric size	39 (92.9)
Straight laryngoscope blade (Miller type)	31 (73.8)
Pediatric size	31 (73.8)
McCoy laryngoscope	0 (0)
Stylet	41 (97.6)
Gum elastic bougie	14 (33.3)
Alternative intubation equipment	
Rigid video laryngoscope^a^	39 (92.9)
Airway scope^®^	33 (78.6)
McGRATH MAC^®^	12 (28.6)
King Vision^®^	1 (2.4)
Airtraq^®^	1 (2.4)
Flexible fiber scope	3 (7.1)
Retrograde intubation kit	1 (2.4)
Surgical airway equipment	42 (100)
Cricothyroidotomy kit	26 (61.9)
Scalpel and hemostat	16 (38.1)
Alternative ventilation equipment^a^	
Supraglottic airway device	7 (16.7)
Pediatric size	5 (11.9)
Intubating laryngeal mask airway	2 (4.8)
Oral airway	21 (50.0)
Pediatric size	16 (38.1)
Nasal airway	36 (85.7)
Pediatric size	8 (19.0)
Device to confirm endotracheal intubation^a^	
Capnometry	28 (66.7)
Esophageal detector	7 (16.7)
Any other devices	4 (9.5)
Packaging unit containing items 1–4	
Complete packaging	16 (38.1)
Partial packaging	15 (35.7)
No packaging	11 (26.2)

Based on the replies of 42 of the 45 HEMS queried

^a^HEMS base hospitals may have more than one of the specified equipment items

**Table 2:** Drugs that facilitate prehospital endotracheal intubation and reversal agents carried by Japanese helicopter emergency medical services (HEMS)

Item	*N* (%)
Analgesics^a^	
Fentanyl	13 (31.0)
Morphine	16 (38.1)
Ketamine	12 (28.6)
Pentazocin	25 (59.5)
Buprenorphine	15 (35.7)
Lidocaine	29 (69.0)
Lidocaine spray	6 (14.3)
Any other analgesic	0 (0)
Sedatives^a^	
Midazolam	39 (92.9)
Diazepam	38 (90.5)
Propofol	10 (23.8)
Thiopental	4 (9.5)
Haloperidol	2 (4.8)
Any other sedatives	0 (0)
Neuromuscular blocking agents^a^	
Rocuronium	19 (45.2)
Vecuronium	18 (42.9)
Pancuronium	0 (0)
Succinylcholine	0 (0)
Any other neuromuscular blocking agents	0 (0)
Reversal agents^a^	
Sugammadex	5 (11.9)
Flumazenil	1 (2.4)
Naloxone	1 (2.4)

Based on the replies of 42 of the 45 HEMS queried

^a^HEMS base hospitals may have more than one drug

**Table 3:** On-board medical members in Japanese helicopter emergency medical services (HEMS)^a^

Item	*N* (%)
*On-board staff members*	*N* = 42
Two physicians and one nurse	6 (14.3)
One physician and one nurse	20 (47.6)
One physician, one nurse, and one on-the-job trainee	16 (38.1)
*Board certification of on-board physicians* ^b^	*N* = 347
Emergency medicine	263 (75.8)
General surgery	54 (15.6)
Intensive care	52 (15.0)
Anesthesiology	35 (10.1)
Cranial surgery	16 (4.6)
Orthopedics	14 (4.0)
Cardiovascular medicine	14 (4.0)
Respiratory medicine	4 (1.2)
Any other board certifications	55 (15.9)
*Certification of on-board nurses* ^b^	*N* = 326
Emergency nursing	58 (17.8)
Intensive care	3 (0.9)
Pediatric emergency nursing	1 (0.3)
Any other certifications	5 (1.5)

^a^Based on the replies of 42 of the 45 HEMS queried

^b^Physicians and nurses may have more than one on-board certification

**Table 4:** Association between the availability of difficult airway management resources specified in the JSA, ASA, and DAS airway management algorithms and the number of dispatches, region, and foundation date in Japanese helicopter emergency medical services (HEMS)

	Number of dispatches (year)			Region			Foundation date		
	*N* (%)			*N* (%)			*N* (%)		
	≥447 *N* = 21	<447 *N* = 21	*p* ^a^	East *N* = 22	West *N* = 20	*p* ^a^	2001–2009 *N* = 19	2010–2015 *N* = 23	*p* ^a^
Can call for help	3 (14.3)	3 (14.3)	1.00	4 (18.2)	2 (10.0)	0.67	2 (10.5)	4 (17.4)	0.67
Supraglottic airway device available	2 (9.5)	5 (23.8)	0.41	5 (22.7)	2 (10.0)	0.41	3 (15.8)	4 (17.4)	1.00
Capnometry available	14 (66.7)	14 (66.7)	1.00	17 (77.3)	11 (55.0)	0.19	13 (68.4)	15 (65.2)	1.00

Based on the replies of 42 of the 45 HEMS queried

*ASA* American Society of Anesthesiologists, *DAS* Difficult Airway Society, *JSA* Japanese Society of Anesthesiologists

^a^ *p* values derived from Fisher’s exact test

## Discussion

In Japan, compliance with standard DAM algorithms [11–13] in prehospital settings is not currently feasible because of the limited availability of back-up personnel and alternative ventilation equipment. Our study showed that additional assistance and SGA were available in <20 % of HEMS bases. Although surgical airway was attainable at all base hospitals, no hospital was able to attain all of the steps. Of note was that only one in ten prehospital physicians were board-certified anesthesiologists. These findings suggest that, in their current form, prehospital settings in Japan do not allow safe ETI. All care providers who participate in prehospital airway management should be aware of the limited human and equipment resources encountered under current working conditions. If the patient is expected to have a difficult airway, ETI should not be attempted in the prehospital setting, except in immediate life-threatening scenarios (i.e., airway obstruction). Otherwise, to avoid a potentially catastrophic situation, oxygen should be optimized and hospital transfer accelerated to obtain prompt access to advanced in-hospital human and equipment resources [10].

### Limitations in the call for help in prehospital settings

More than 85 % of the bases in this study had no back-up personnel. The ‘call for help’ is the first step and the most important aspect of the DAM algorithms [11–13]. Jaber et al. [27] recently reported that having two care providers present was a vital element for successful ETI of critically ill patients. Limited help is one of the greatest disadvantages of HEMS and the situation most unlike that of a hospital. DAM in the poorly prepared prehospital setting can lead to serious adverse events regarding patient care. Past reports from outside Japan have shown increases in ETI difficulty [3–6] and severe ETI-related complications [28, 29] when the procedure is performed outside hospitals. In fact, under these high-risk conditions ETI should not be attempted if manual ventilation is successful. Paal et al. [10] also emphasized the importance of avoiding repeat ETI attempts in prehospital settings. According to the best available evidence, prehospital ETI does not provide any survival benefits for patients suffering out-of-hospital cardiac arrest [30, 31], traumatic brain injury [32–34], or multiple traumas [35–37]. These patients are the most vulnerable to the detrimental cardiovascular effects of the positive pressure breaths delivered through an endotracheal tube [38]. Davis et al. [32] showed that, even after adjusting for multiple clinical variables affecting outcome, prehospital intubation was associated with decreased survival among patients with moderate to severe traumatic brain injury. Warner et al. [34] found a correlation between patients with severe traumatic brain injury who received prehospital rapid sequence intubation (RSI) and both mortality and hyperventilation (arterial PCO_2_ < 24 mmHg). Shafi et al. [35] demonstrated that prehospital ETI in trauma patients is associated with decreased survival, possibly because of positive pressure ventilation during the hypovolemic state. Stockinger et al. [36] found that prehospital ETI conferred no survival benefit over bag valve mask ventilation and increased prehospital time. These studies, together with our own, suggest that the threshold for ETI outside the hospital should be higher than in the hospital emergency room. At least in Japan, if not elsewhere, the evidence underlines the need for a deliberation of the risks and benefits of prehospital ETI.

### Neglect of the importance of SGA as a rescue ventilation device in prehospital settings in Japan

In this study, SGA was available in only 16.7 % of the prehospital settings, and a pediatric-sized device in only 11.9 %. In Europe, SGA is available in 92.0–97.6 % of prehospital settings [19, 20]. Thus, in Japan, SGA has been undervalued as a rescue ventilation device in prehospital settings. Each HEMS must have back-up ventilation strategies [11–13] because the consequences of failed intubation can be devastating. SGAs have several advantages for use in rescue ventilation [11–13] and should be available wherever anesthesia is carried out in the prehospital setting [15, 16]. Lockey et al. [39] and Combes et al. [40] reported that all patients whose tracheas could not be intubated in a prehospital emergency setting were successfully rescued by SGA. Our study showed that prehospital airway equipment was arbitrarily selected by each base. However, its standardization, including a SGA or other rescue ventilator device would be beneficial.

Successful SGA insertion is related to operator experience [41]. In a CVCI scenario, which is a definite opportunity to use SGA [11–13], the victim is at high risk of cardiac arrest due to hypoxemia. Therefore, appropriate training in SGA insertion is crucial for health care professionals who are likely to participate in airway management. Nevertheless, other than elective surgery, the clinical settings in which patients are ventilated with SGA are relatively rare. To gain SGA insertion experience and airway management competence, HEMS physicians should participate in a certain number of these procedures in the hospital operating room [42, 43]; this is especially important for those whose clinical background is not anesthesiology. Thus, as in other countries [42, 43], airway management training programs in the operating room for HEMS physicians should be established throughout Japan.

### Limited availability of capnometry in prehospital settings in Japan

Capnometry was available in approximately two-thirds of the HEMS bases surveyed. By contrast, capnometry is available in 85–100 % of the prehospital settings in Europe [19, 20]. Verification of endotracheal tube placement is an indispensable part of any DAM strategy [11–13], and capnometry is both more sensitive and more specific than auscultation alone in recognizing correct tube placement following emergency intubation [44–46]. Continuous end-tidal carbon dioxide (ETCO_2_) monitoring is also useful to detect inadvertent tube dislodgement during patient transport [47]. Silvestri et al. [47] reported that when paramedics used continuous ETCO_2_ monitoring in prehospital settings, there were no cases of unrecognized misplaced intubation in patients upon emergency room arrival, whereas the misplaced intubation rate was 23 % when continuous ETCO_2_ was not used. A national audit in the UK [14] found that failure to use capnometry in treating a difficult airway probably contributed to at least some of the fatal outcomes. ETCO_2_ confirmation of tube placement and continuous monitoring of the endotracheal tube position are now a standard of care in the operating room [11–13] and in the intensive care unit [14]. As a result, the use of ETCO_2_ monitoring has become an important aspect of emergency medicine [14, 47]. The incorporation of ETCO_2_ confirmation and continuous monitoring into out-of-hospital airway management would therefore improve patient management by prehospital health care professionals.

### Shortage of board-certified anesthesiologists as prehospital physicians in Japan

According to our survey, board-certified anesthesiologists comprised only 10.1 % of all prehospital physicians in Japan. In Scandinavia and Germany, by contrast, prehospital airway management is mostly performed by anesthesiologists with specific prehospital training [15, 17, 48, 49]. As concluded by Lockey et al. [15] and clearly stated in the prehospital advanced airway guidelines of Scandinavia [17], the providers of prehospital airway management should have the same level of competence as in-hospital anesthesia providers. To date, standard airway management competence for HEMS physicians in Japan has not been defined. Breckwoldt et al. [6] investigated the incidence of difficult ETI (number of ETI attempts >3) in the prehospital setting, comparing emergency physicians with a clinical background in anesthesiology (expert status) and those with a background in internal medicine. They found an association between expert status and a significantly lower incidence of difficult ETI and thus proposed that the value of day-to-day ETI experience be considered in the treatment of a difficult airway outside the hospital. As we pointed out in a previous study, the skill and knowledge of anesthesiologists should be fully employed for high-risk ETI rather than limited to the operating room [50]. To improve prehospital airway management in Japan, more anesthesiologists are recommended to participate in prehospital medical care. There is however a lack of anesthesiologists in Japan, and the regular training of HEMS (non-anesthesiologist) physicians in the operating room would also be beneficial for airway training and to gain experience [42, 43]. For the retention of ETI skills, HEMS physicians should be required to perform a certain number of procedures within a defined period [51].

### Preparedness of HEMS to perform surgical airway management

While all bases had surgical airway devices, few had reversal agents. This finding probably reflects the fact that in the field of emergency medicine, even if difficulties are encountered, waking a patient following RSI is rare [39, 52], because a patient requiring emergency ETI is absolutely in need of a definitive airway. In these settings, a timely surgical airway may be life-saving [53] and more important than waking the patient. Previous studies reported an incidence of prehospital cricothyroidotomy of 0.5–2.4 % [39, 54, 55], compared with 0.005–0.025 % [56] in the operating room. The need for an emergency surgical procedure was 100-fold higher in prehospital settings than in the hospital operating room. All HEMS physicians therefore must be proficient in this alternative intubation technique. To maintain their proficiency, they should receive regular off-the-job training in, for example, the use of a simulator [57, 58].

### Recommendations from this study

This study revealed that the limited availability of back-up personnel, alternative ventilation, and confirmation equipment in prehospital settings in Japan greatly hinders DAM. Given the current situation in Japan, rapid transport is preferable over active airway management in the field if ventilation and oxygenation are acceptable. Avoiding a prehospital ETI attempt is particularly important if a difficult airway is anticipated [10]. As stated by the Scandinavian Society for Anaesthesiology and Intensive Care Medicine [17], “Even for maximally skilled personnel, it should always be considered whether ETI attempts should be performed pre-hospitally or be postponed till more advanced in-hospital techniques are available.” Nevertheless, ‘forced to act' scenarios may arise despite an anticipated difficult airway [59]. Examples include a patient with immediate or deteriorating airway obstruction or a patient whose oxygenation is unacceptable even after manual bag mask ventilation. In these cases, multiple ETI attempts should be strictly prohibited and a rescue technique, including a surgical airway, should be performed without hesitation because (1) multiple ETI attempts in a setting of limited human and equipment resources are known to increase the risk of severe complications [7–9, 14, 28, 29] and (2) complications in the management of a difficult airway can increase prehospital time, which is associated with an adverse outcome [60–62]. Thus, in prehospital settings, a difficult airway should be managed in a time-sensitive manner [59]. There is a tendency for laryngoscopists to persist with an ETI even if it is proving to be difficult [63]; this inevitably results in the delayed implementation of alternative intubation techniques. However, any hesitancy regarding the latter will be readily overcome once proficiency with an alternative rescue technique is acquired [57].

In Japan, prehospital airway equipment is not standardized; it is selected at the discretion of the manager of each base. To ensure homogeneous prehospital airway strategies, the equipment carried out-of-hospital needs to be standardized and should be consistent with that of a hospital operating room [14]. Suggestions for DAM resources have been proposed by the JSA [11], ASA [12], and DAS [13] which include rigid laryngoscope blades of alternate design and size from those routinely used, video laryngoscope, tracheal tubes of assorted sizes, tracheal tube guides including a stylet and a gum elastic bougie, noninvasive airway ventilation equipment including assorted sizes of SGA and nasal/oral airway, equipment suitable for emergency invasive airway access, an exhaled carbon dioxide detector, and a portable storage unit containing these devices.

Adequate experience and the training of every HEMS physician in the use of this equipment are absolute requirements. Airway management training programs for HEMS physicians [42, 43] that include sufficient ETI and SGA caseloads in the operating room should be available throughout Japan. Regular off-the-job training can aid in maintaining the skills needed for surgical airway management [57, 58].

### Study limitations and advantages

There were two major limitations to this study. First, our survey did not determine the frequency of difficult airways and CVCI situations, nor did it obtain information on airway management practices in prehospital settings. The optimal management of difficult airway situations despite the limited resources of HEMS bases remains to be determined in future works. Second, because our questionnaire was self-administered, there may have been reporting bias. Nonetheless, in our survey of HEMS hospitals in Japan, the response rate was extremely high (42 of 45). Our study thus provides an accurate depiction of the current state of prehospital advanced airway management in Japan but it also reveals the areas in need of improvement.

## Conclusion

In Japan, compliance with standard airway management algorithms is currently not practicable [11–13] in prehospital settings, given the limited availability of alternative ventilation equipment and back-up personnel. Because the prehospital setting in Japan is not conducive to successful DAM, all healthcare professionals working in this environment should seriously consider whether ETI should be performed or whether the more prudent decision is to postpone the procedure until more advanced in-hospital techniques and an adequate number of personnel are available. In addition, the airway equipment, alternative ventilation equipment, and confirmation device carried out of hospital should be standardized. Because adequate experience is essential in the successful management of challenging situations, airway management training programs for HEMS physicians should be made available throughout Japan.

## Electronic supplementary material

Below is the link to the electronic supplementary material.

Supplementary material 1 (DOCX 24 kb)

Supplementary material 2 (DOCX 24 kb)
