# Supplementary material for: Are prehospital airway management resources compatible with difficult airway algorithms? A nationwide cross-sectional study of helicopter emergency medical services in Japan
Source: J Anesth. 2015 Dec 29;30:205–14. doi: 10.1007/s00540-015-2124-7 (PMC4819484; doi:10.1007/s00540-015-2124-7)
Supplement: Supplementary file 1 — Supplementary material 1 (DOCX 24 kb) [file 540_2015_2124_MOESM1_ESM.docx]

**Airway equipment and drugs in helicopter emergency medical services in Japan: A questionnaire**

| **Note**   - Please fill out this form based on the situations of your hospital in May 2015, unless any annotations are provided. |
| --- |

| **Hospital name** | |  | | | |  |  |
| --- | --- | --- | --- | --- | --- | --- | --- |
| 1. How many beds does your hospital have? | | | |  | | | |
| 1. How many ambulances did your hospital receive in 2014? | | | |  | | | |
| 1. How many HEMS dispatches in 2014? | | | |  | | | |
| 1. Is the following airway equipment available in the prehospital setting?   **(1) Direct laryngoscope and intubation adjuncts** | | | | | | | |
| 1. **Direct laryngoscope and adjunct**   **(a)** Curved laryngoscope blade (Macintosh type)  **(b)** Pediatric size | | | | **□ Yes □ No**  **□ Yes □ No** | | | |
| 1. Straight laryngoscope blade (Miller type)   **(a)** Pediatric size | | | | **□ Yes □ No**  **□ Yes □ No** | | | |
| 1. McCoy laryngoscope | | | | **□ Yes □ No** | | | |
| 1. Stylet | | | | **□ Yes □ No** | | | |
| 1. Gum elastic bougie | | | | **□ Yes □ No** | | | |
| **(2) Alternative intubation equipment** | | | | | | | |
| 1. Rigid video laryngoscope | | | | **□ Yes □ No** | | | |
| 1. Please provide the product name.   **(a)** If you have more than 1 rigid video laryngoscope, please enumerate. | | | |  | | | |
| 1. Flexible fiber scope | | | | **□ Yes □ No** | | | |
| **(3) Alternative ventilation equipment** | | | | | | | |
| 1. **Supraglottic airway**   **(a)** Pediatric size  **(b)** Intubating laryngeal mask | | | | **□ Yes □ No**  **□ Yes □ No**  **□ Yes □ No** | | | |
| 1. Oral airway   **(a)** Pediatric size | | | | **□ Yes □ No**  **□ Yes □ No** | | | |
| 1. Nasal airway   **(a)** Pediatric size | | | | **□ Yes □ No**  **□ Yes □ No** | | | |
| **(4) Surgical airway device** | | | | | | | |
| 1. Cricothyroidotomy kit | | | | **□ Yes □ No**  **□ Only scalpel and hemostat** | | | |
| 1. Retrograde intubation kit | | | | **□ Yes □ No** | | | |
| **(5) Device to confirm endotracheal intubation** | | | | | | | |
| 1. Capnometry | | | | **□ Yes □ No** | | | |
| 1. Esophageal detector | | | | **□ Yes □ No** | | | |
| 1. Any other devices | | | |  | | | |
| **(6) Packaging unit** | | | | | | | |
| **Packaging unit containing (1)–(5)** | | | | **□ Yes □ No**  **□ Partially** | | | |
| **5.** Are the following drugs available in the prehospital setting?  **(1) Analgesic drugs** | | | | | | | |
| **□** Fentanyl  **□** Pentazocine | | | | **□** Morphine  **□** Buprenorphine | | | |
| **□** Lidocaine spray  **□** Lidocaine | | | | **□** Tramadol  **□** Ketamine | | | |
| **□** Any other analgesic drugs (please specify) | | | |  | | | |
| **(2) Sedatives** | | | | | | | |
| **□** Midazolam  **□** Propofol | | | | **□** Diazepam  **□** Thiopental | | | |
| **□** Droperidol | | | | **□** Haloperidol | | | |
| **□** Any other sedative drugs (please specify) | | | |  | | | |
| **(3) Neuromuscular blocking agents** | | | | | | | |
| **□** Succinylcholine  **□** Vecuronium | | | | **□** Rocuronium  **□** Pancuronium | | | |
| **□** Any other neuromuscular blocking agents  (please specify) | | | |  | | | |
| **(4) Reversal agents** | | | | | | | |
| **□** Sugammadex  **□** Naloxone | | | | **□** Flumazenil | | | |
| **□** Any other reversal agents (please specify) | | | |  | | | |
| **(5) Any other drug to facilitate endotracheal intubation** | | | |  | | | |
| **If available, please specify.** | | | |  | | | |
| **(6)** **Members of the on-board staff** | | | | | | | |
| A. Who are the members?  e.g., Physician 1, Nurse 1, and On-the-job trainee 1 | | | |  | | | |
| **(2) On-board physicians** | | | |  | | | |
| **A.** How many prehospital physicians does your hospital have? | | | |  | | | |
| **B.** Of these, how many are board-certified in the fields listed below?*  *Physicians may have more than one board certification. | | | | | | | |
| Emergency medicine | |  | | | Anesthesia |  | |
| Intensive care | |  | | | General surgery |  | |
| Orthopedics | |  | | | Cranial surgery |  | |
| Cardiovascular medicine | |  | | | Respiratory medicine |  | |
| Any other board certifications | |  | | |  | | |
| **(3) On-board nurses** | | | | |  | | |
| **A.** How many prehospital nurses does your hospital have? | | | | |  | | |
| **B.** Of these, how many are board-certified in the fields listed below? | | | | | | | |
| Emergency nursing | |  | | Intensive care | |  | |
| Perioperative nursing | |  | | Pediatric emergency nursing | |  | |
| Any other board certifications | |  | |  | | | |

| **Free comment** |  |
| --- | --- |

**Thank you very much for your time and collaboration.**
